# Quinine plus clindamycin vs artemether-lumefantrine for treatment of uncomplicated falciparum malaria in western Kenya

**DOI:** 10.1186/s12936-022-04222-6

**Published:** 2022-06-21

**Authors:** Charles O. Obonyo, Bernhards R. Ogutu

**Affiliations:** 1 Centre for Global Health Research, Kenya Medical Research Institute, Kisumu, Kenya; 2 Centre for Clinical Research, Kenya Medical Research Institute, P.O. Box 20778, Nairobi, Kenya

We read with interest the comment by Kremsner and Krishna on our paper published on this Journal. The comment correctly points out that we documented an adequate clinical and parasitological response (ACPR) of 44% with quinine plus clindamycin compared with 97% on artemether-lumefantrine assessed on day 28 after starting treatment. We recognize the long-standing research interest by Kremsner and Krishna on the role of clindamycin-based combinations in the treatment of falciparum malaria, demonstrated by their publications. In this comment, Kremsner and Krishna are concerned with the level of ACPR on the quinine plus clindamycin arm in our study. They cite a few of their studies conducted in Gabon and Brazil that evaluated the efficacy of a 3-day course of 12-hourly quinine plus clindamycin treatment for participants with either uncomplicated or severe malaria. In the cited studies, administration of a relatively higher dose of quinine (15 mg/kg bd) combined with a relatively lower dose of clindamycin (7 mg/kg bd) was consistently associated with cure rates of between 88 and 100% (excluding reinfections). They correctly observe that 54% of children treated with quinine plus clindamycin in our study were still parasitaemic on day 3. However, they confirm that the mean parasite clearance with quinine plus clindamycin was slow (48 to 65 h) even in their previous studies and they commonly observed that up to 50% of participants were still parasitaemic on day 3 but “without the need for rescue treatment”. In conclusion, Kremsner and Krishna highlight the continuing need to evaluate non-artemisinin-based combinations for the treatment of uncomplicated malaria using appropriate study designs and correct dosages to avoid exposing participants to undertreatment and possible misinterpretation of the findings.

We do not agree with the statement by Kremsner and Krishna that our study was probably not well-designed, which they have not elaborated. We followed the standard methods for the design of randomized controlled parallel-group clinical trials for assessing the treatment efficacy of anti-malarials [1–6]. Specifically, we implemented an open-label, randomized controlled trial to evaluate the efficacy of quinine plus clindamycin vs artemether-lumefantrine for treatment of Kenyan children (under 5 years) with uncomplicated falciparum malaria. We pre-specified the eligibility criteria, endpoints and duration of follow-up in the protocol, supervised the administration of study treatment and used objective outcome measures to assess treatment efficacy. Our results are internally valid as we minimized the possibility of systematic bias by ensuring that our participants had comparable prognosis (by randomization), had comparability of treatments (by using oral 12-hourly treatment for 3 days) and comparability of outcome information (by using hard outcomes) [7]. The possibility of information bias (from attrition) was minimized by intention-to-treat analysis. We are not convinced that we undertreated the participants on the quinine plus clindamycin arm of our study. Lell and Kremsner 2002, reviewed 13 studies (only 3 were in children) that had evaluated the efficacy of quinine (8–12 mg/kg bd) plus clindamycin (5–10 mg/kg bd) in the treatment of adults or children with uncomplicated falciparum malaria and found cure rates of 88–100% [8]. We used a similar range of dosages in our study but found comparatively low cure rates with this regimen. We speculated that the low unexpected cure rates following treatment with quinine plus clindamycin could be explained by the short treatment course (3 days), the low quinine (10 mg/kg bd) dose, a declining quinine efficacy or the slow action of clindamycin. We do not agree with the conclusion of Kremsner and Krishna that persistent parasitaemia at day 3 is not a useful marker for assessing treatment success or failure. In our study, half of the children treated with quinine plus clindamycin were still parasitaemic by day 3 post-treatment. This was not a surprising finding as slow parasite clearance is an expected phenomenon with clindamycin treatment thought to derive from “delayed parasite death” [9]. Delayed clearance of malaria parasites by the third day after treatment has consistently been found to strongly correlate with anti-malarial treatment failure [10–13].

Overall, all the studies cited by Kremsner and Krishna had methodological flaws, had used a 3-day 12 hourly regimen of quinine plus clindamycin with comparable doses, long mean parasite clearance times but higher cure rates. It is important to note that there were probably spatio-temporal differences in parasite resistance patterns between Gabon and western Kenya. Only one of the cited studies was closely comparable to our study because they used a well-designed study to compare the efficacy of quinine plus clindamycin to an artemisinin-based combination therapy (artesunate plus clindamycin) in the treatment of African children with uncomplicated falciparum malaria [14]. In conclusion, the weight of the available evidence does not support the recommendation of quinine plus clindamycin for the treatment of children with uncomplicated falciparum malaria.
